# Tertiary lymphoid structures in autoimmune diseases

**DOI:** 10.3389/fimmu.2023.1322035

**Published:** 2024-01-08

**Authors:** Yuanji Dong, Ting Wang, Huaxiang Wu

**Affiliations:** ^1^ Department of Rheumatology, The Second Affiliated Hospital of Zhejiang University School of Medicine, Hangzhou, Zhejiang, China; ^2^ Department of Respiratory Disease, Thoracic Disease Center, The First Affiliated Hospital, College of Medicine, Zhejiang University, Hangzhou, Zhejiang, China

**Keywords:** tertiary lymphoid structures, autoimmune disease, fibroblasts, immune cells, potential target

## Abstract

Tertiary lymphoid structures (TLSs) are organized lymphoid-like aggregations in non-lymphoid tissues. Tissues with chronic and persistent inflammation infiltration may drive and form ectopic germinal center-like structures, which are very common in autoimmune diseases, chronic infections, and tumor microenvironments. However, the mechanisms governing the formation of TLSs are still being explored. At present, it is not clear whether the formation of TLSs is associated with local uncontrolled immune inflammatory responses. While TLSs suggest a good prognosis in tumors, the opposite is true in autoimmune diseases. This review article will discuss the current views on initiating and maintaining TLSs and the potential therapeutic target in autoimmune diseases.

## 1 Introduction

The immune system is an efficient weapon for the body to fight against foreign invaders, of which adaptive immunity provides enhanced defense against specific pathogens. T and B lymphocytes are the core members of adaptive immunity, and they are both generated in the bone marrow. T cells enter the lymphatic system after a rigorous selection (immune tolerance) in the thymus, while B cells are in the bone marrow. Naive T and B cells circulate mainly in secondary lymphoid organs. Upon infection, lymphocytes are activated and exert an effective immune response. Activated plasma cells secrete antibodies and activated T cells egress into the infected tissues. Under normal circumstances, after the infection is cleared, immune cell (mainly tissue-resident macrophages) by switching their phenotype from pro- to anti-inflammatory/pro-healing plays a pivotal role in tissue repair and metabolic homeostasis (1). Activated T cells will undergo apoptosis via activation-induced cell death (AICD) (2). However, in the case of antigen persistence, such as rheumatic diseases (3), solid tumors (4, 5), organ transplantation (6), chronic infections (4), etc., the situation may be different. T and B cells as well as stromal cells in the chronic inflammatory microenvironment create the conditions for the formation of TLSs in tissues (7, 8).

TLSs mainly consist of highly organized clusters of lymphocytes formed not in secondary lymphoid organs (SLOs) (9). Firstly, this structure is non-encapsulated, secondly, there is a clear separation of T and B cells, in which B cells are located in the core of the follicle, the periphery is a T cell-rich area, and the outermost is plasmablast and plasma cells (10). In addition, this structure can also contain high endothelial venules (HEV) and stromal cell-derived follicular dendritic cells. However, cell types and molecular signals involved are different during the initiation of TLSs (11, 12). Nevertheless, some chemokines related to the segregation of T and B cells and some enzymes involved in the function of the germinal center such as activation-induced cytidine deaminase (AID) are the same (11, 12). These studies provided useful information for TLSs-targeted therapies. In this review article, we mainly explore the initiating factors and characteristics of TLSs in immune-related diseases, including Rheumatoid Arthritis, Sjogren’s Syndrome, Lupus Nephritis, IgG4-related Sialadenitis, Myositis, Type 1 Diabetes, and Multiple Sclerosis. We also explore the relationship between TLSs and disease severity, and the potential for TLSs-targeted therapies.

## 2 SLOs and TLSs development

**Figure 1:**
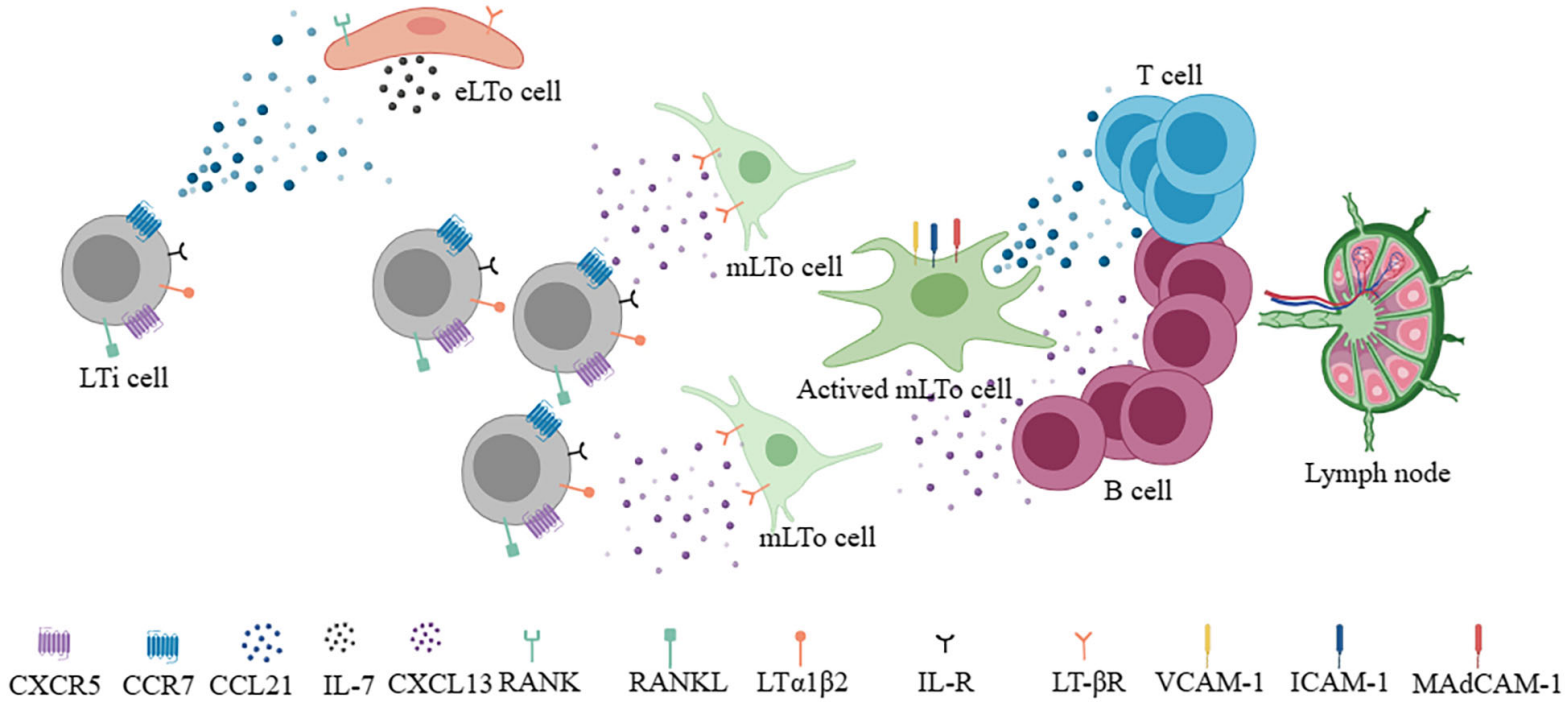
During the development of lymph nodes, LTi cells interact with eLTo cells and mLTo cells to secrete chemokines (CCL21, CXCL13) and survival factors (IL-7), respectively and form different T and B compartments to develop into functional lymph nodes.

To understand the neogenesis of TLSs, it is first necessary to understand the developmental processes of SLOs (13). In terms of lymph nodes, two cell types play an important role in the process of formation, lymphoid tissue inducer (LTi) cells and lymphoid tissue organizer (LTo) cells (14–16). The former namely CD45+CD4+CD3- innate lymphoid cells are characterized by RORgt and Id2 transcription factor (17). The latter includes endothelial lymphoid tissue organizer cells (eLTo) and mesenchymal lymphoid tissue organizer cells (mLTo). Initially, LTi cells migrate from the fetal liver into the lymph node site, where blood flow and lymph flow intersect. At this time, chemokine ligand 21 (CCL21) and IL-7 secreted by eLTo cells act on LTi cells which express chemokine receptors 7 (CCR7) and IL-7R, promoting the upregulated of lymphotoxin a1β2 (LTa1β2) (13). At the same time, LTi cells expressing receptor activator of NF-KappaB ligand (RANKL) and LTa1β2 act on eLTo cells, which express RANK and LTβ receptors, resulting in the secretion of CCL21 and IL-7 to further promote the recruitment of LTi cells. CXCR5+ LTi cells can also interact with CXCL13+ mLTo. The interaction between LTa1β2+ LTi and ILβR+ LTo promoted the expression of homeostatic chemokines CCL19, CCL21, CXCL13, pro-survival factor IL-7, and adhesion molecules vascular cell adhesion molecule 1 (VCAM-1), intercellular adhesion molecule 1 (ICAM-1), mucosal addressin cell adhesion molecule 1 (MadCAM-1) and peripheral lymph node addressin (PNAd) (18, 19). These molecules regulate the subsequent recruitment of immune cells and the vascularization of high endothelial venule (HEV). CCR7+ T cells and CXCR5+ B cells are guided by CCL19+/CCL21+ FRCs and CXCL13+ FDCs to form T and B cell zones (18) (**Figure 1**). Notably, CXCR5+B cells could induce LTa1β2 expression. In addition, CXCL12+ stromal cells and CXCL13+ FDC can also mediate B cell affinity maturation. Altogether, the formation of SLOs involves the interaction between hematopoietic cells and non-lymphoid stromal cells, secreting of cytokines (ILα1β2, IL-7, etc.), chemokines (CCL19, CCL21, CXCL13, etc.), adhesion molecules (VCAM-1, ICAM-1, Mad-CAM1, PNAd, etc.), and eventually forming and maintaining the lymphoid niche. Although the development of TLSs is still not fully understood, evidence supports that many immune cells (B cells, Th17, ILC3, macrophages, etc.) can potentially replace LTi cells (20–23). Also, some local stromal cells and immune populations (such as synovial fibroblasts, fat cells, vascular smooth muscle cells, etc.) can act as LTo cells (8, 24, 25). In contrast, the roles of chemokines and adhesion molecules in SLO and TLSs are similar. Therefore, under the stimulation of chronic inflammation or immune microenvironment, the gradual formation of chemokines and adhesion molecules is similar to the lymphoid niches, which promotes the generation of TLSs. Local immune cells such as B cells or macrophages act as LTi, stimulating IL-βR signaling on stromal cells to promote VEGFC secretion and HEV formation (26). Different levels and types of chemokines and adhesion molecules shape different degrees and components of TLSs.

## 3 Composition and function of TLSs

The main cell subtypes in the TLSs were T and B cells, in which B cells are located in the core and these structures are similar to germinal centers. These B cells may cluster around CD21+ FDCs, which are of mesenchymal origin and capture and present antigens to B cells (27). In contrast, T cells and CD83+ DCs are located in the periphery and among them are interspersed with CD68+ macrophage, which collects around dense reticular cells (similar to FRCs) (28). In addition, HEVs expressing PNAd (peripheral lymph node addressin protein) and L-selectin ligands can also be found in TLSs, ensuring that blood lymphocytes can be recruited (29, 30). Since TLSs perfectly mimic the formation of SLOs, can they also functionally achieve B cell activation and antibody production locally? Indeed, T and B cells can interact with each other in TLSs and promote local antibody production, and AID enzyme expression demonstrates the class switching and affinity maturation of B cells (31). In addition to autoimmune diseases, TLSs can also combat infection locally by antigen-specific B cells in chronic infections and can achieve clonal expansion, somatic hypermutation, and isotype conversion in tumors (32–34). Particularly, in the TLSs of renal cell carcinoma, B cells can locally mature into IgG+ plasma cells, which produced IgG can label tumor cells and induce apoptosis (35). However, we must notice that not all TLSs examined are mature and functional. A study of lung squamous cell carcinoma-related TLSs found that B and T cell aggregates represent the initial stages of TLSs, FDC differentiation represents primary follicle-like TLSs (PFL-TLSs), and T, B, and DC reaction with local antigens represent the secondary follicle-like TLSs (SFL-TLSs). They can also be distinguished by immunohistochemistry. TLSs lacking CD 21 and CD 23 signals are immature, only CD21 positive are PFL-TLSs, and both CD21 and CD23 positive are SFL-TLSs (36). Subsequent studies further confirmed, that TLS, especially that containing germinal centers and participating in the local reaction has a much lower risk of colorectal carcinoma recurrence, and could predict immune checkpoint inhibitor efficacy in some solid tumors (37, 38). These results suggest that it is not enough to describe whether TLSs exist, it is more meaningful to study the functional status. In addition to effectively presenting the self-antigens, pathogen antigens, or tumor antigens, some studies have also shown the immunosuppressive effect of TLSs. For example, in atherosclerosis or adenocarcinoma models, TLSs-associated Treg may play an immunosuppressive role, which inhibits the development of atherosclerosis and promotes tumor progression (39, 40). These results also suggest that the role of TLSs is also closely related to the cell subtypes and the immune microenvironment. Immune cells exhibit high plasticity, and the change in cytokine environment and cell subtypes could be therapeutic strategies.

## 4 Extrinsic factors and intrinsic components in TLSs

**Figure 2:**
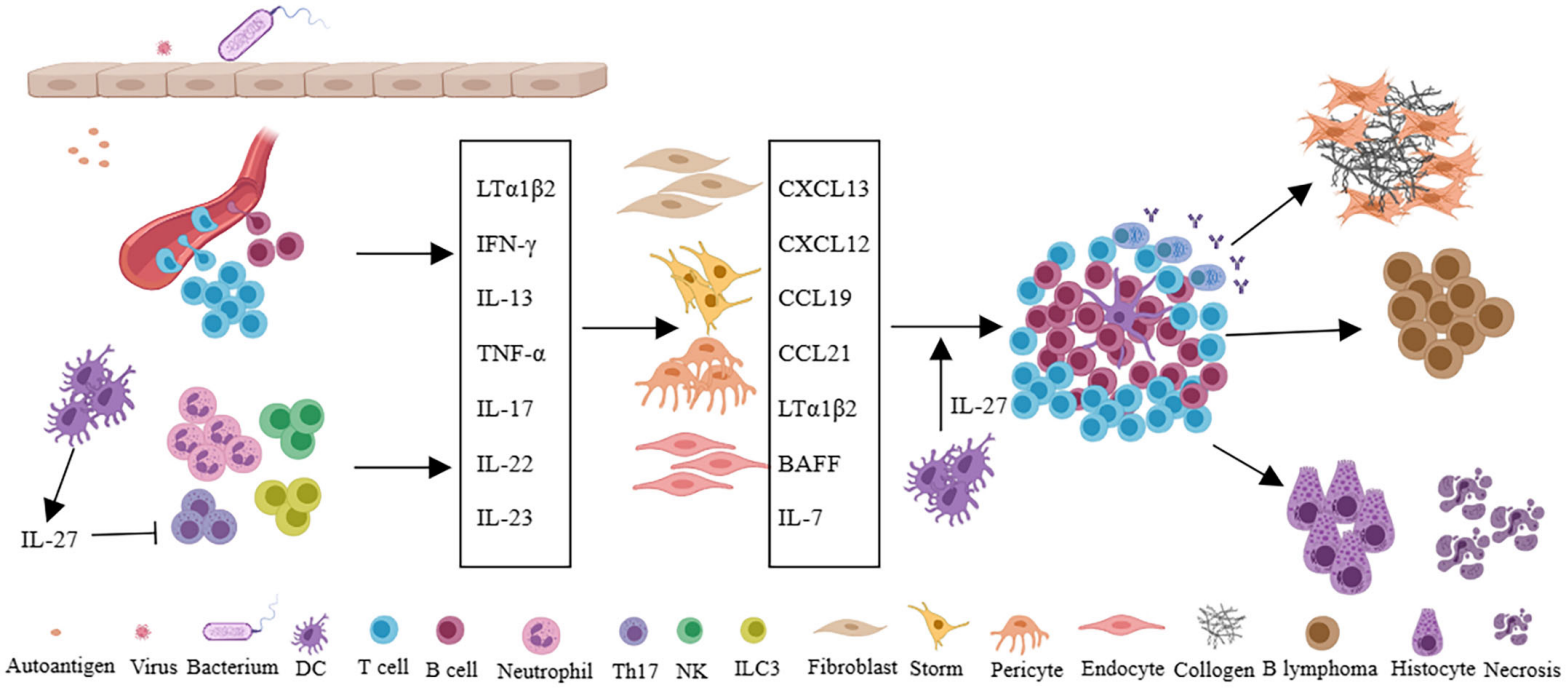
Persistent infection of pathogen or long-term stimulation with autoantigens leads to changes of local microvascular adhesion molecules in tissues, recruitment of immune cells, and secretion of a large number of cytokines such as LTα1β2, IFN-γ, IL-13, TNF-α, IL-17, IL-22, IL-23, et al. Furthermore, immune cells interact with fibroblasts, stromal cells, pericytes, or endothelial cells in local tissues to secrete chemokines (CXCL13, CXCL12, CCL19, CCL21) and cytokines (LTα1β2, BAFF, IL-7), etc., and eventually forms TLSs in local tissues. In autoimmune diseases, TLSs can promote tissue damage and fibrosis, and in specific diseases such as Sjogren’s syndrome, TLSs are also associated with lymphoma. IL-27 can inhibit the formation of TLSs by restricting Th17 and also support the function of TLSs by promoting the production of IL-21 and the function of Tfh cells.

Virus infection, as an important extrinsic factor, is closely related to the formation of TLSs (31, 41). Viral infection often leads to the activation of antiviral immunity, in which Type 1 interferon was induced. The interferon not only inhibits viral replication, but also can promote DC activation and maturation, enhance NK and CD8 + T cell killing ability, and promote the B cells to produce antibodies (42). Viral infection increases the probability of molecular mimicry and autoantigen recognition, and repeated infection can lead to epitope spreading (43). Moreover, virus infection often invades the tissue barrier, leading to epithelial damage, which leads to lymphocyte aggregation and induces resident fibroblasts to undergo phenotypic and functional changes, which provides the basis for the formation of TLSs. The relationship between viral infection and TLSs in Sjogren’s syndrome (SS) has been reviewed (41). Internally, multiple molecular and cellular components have been implicated in the development of TLSs (9, 11, 12). The development of SLOs requires the interaction of LTi and LTo cells. The difference is that LTi cells are not necessary for the formation of the TLSs in mammals (44). A variety of immune cells are thought to act as LTi, such as the activation of T, B, and NK cells, etc (20–23, 45–48). These cells express LTα1β2 and interact with LTβR on stromal cells. During the formation of TLSs, stromal cells, such as gp38+ fibroblasts, act as LTo cells (49). In addition, IL-17-secreting cells such as ILC3, Th17, γδT cells, NK cells, and neutrophils can provide a local inflammatory microenvironment for the initiation of TLSs (9). Dendritic cells and CD68 + myeloid cells by expressing the LTβR also involved in TLSs (50, 51). In addition to different cell types, chemokines, and cytokines also play important roles in the formation and maintenance of TLSs. Upregulation of CCL19 and CCL21 can recruit CCR7+ T and DC cells to tissues, while CXCL12 and CXCL13 attract CXCR4+ and CXCR5+ B cells. Of note, some cytokines or receptors are considered to play an important role in the TLSs of specific tissues or diseases. LTα expression in tumor cells leads to the formation of intratumoral TLSs (52). IL-17 is thought to be associated with TLSs in the bronchi and central nervous system (45, 53). IL-22 has been implicated in TLS in a mouse model of virus-induced SS (54). Activation of LTβR and TNFRI is associated with aortic TLSs (55, 56). Besides positively regulated factors, IL-27 is thought to be negatively correlated with the formation of TLSs (57, 58), despite IL-27 can also promote IL-21 production to maintain and promote the germinal center (59). Moreover, IL-6, IL-7, IL-23, and TNF-α are all associated with TLSs (12) (**Figure 2**).

## 5 Characteristics of TLSs in autoimmune diseases

**Figure 3:**
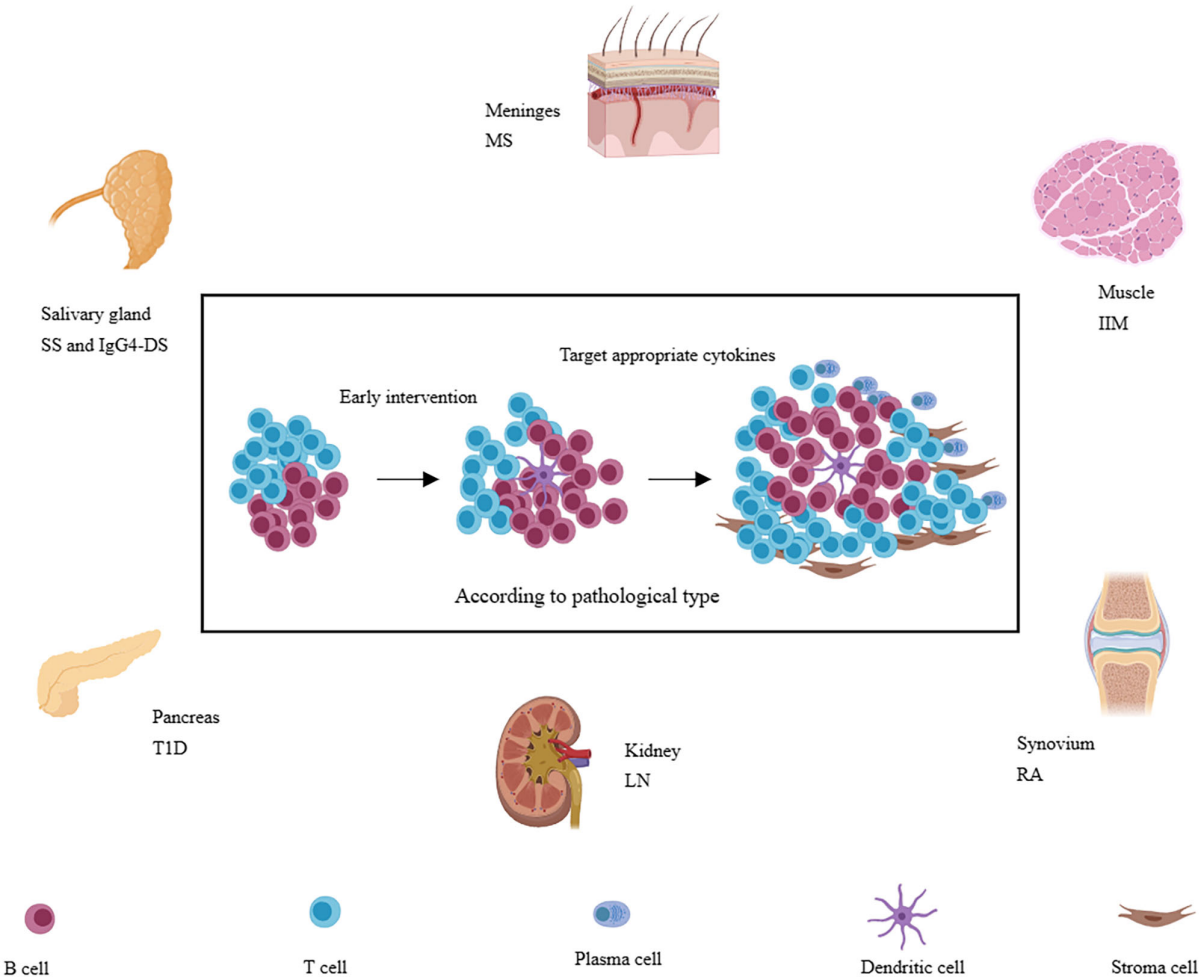
A variety of immune-related diseases, including RA, SS, LN, IgG4-SD, IIM, T1D, and MS et al., can form TLSs in local tissues (synovial membrane, salivary gland, kidney, muscle, islet, and meninges, etc.) and TLSs are also associated with more severe disease phenotypes. Targeting appropriate cytokines may be more effective according to pathology and at the early stage of TLSs formation.

In autoimmune diseases, there often exists persistent chronic inflammation in the affected tissue. However, not all patients with the same disease have developed TLSs. It was reported that the TLSs are preferred to be located in organs or tissues not predisposed embryologically to allow the development of lymphoid tissues, such as the meninges, salivary glands, kidneys, blood vessels, heart, synovium, pancreas, or liver (9, 60), perhaps due to the inefficient presentation of local antigens to the lymphatic draining lymph nodes. The body needs the local lymphoid structure to improve efficiency and increase the defensive intensity. It also suggests that immune tolerance is relatively weak in these tissues. Infiltration of immune cells is more likely to respond to autoantigens. On the other hand, not all patients with autoimmune diseases have this structure, suggesting that the formation of this structure also requires multiple factors. In the following, we will explore the characteristics of TLSs in several common autoimmune diseases (**Figure 3**). We will also describe the incidence, molecular and cell types involved, and function of TLSs in the following diseases (**Table 1**).

**Table 1:** The histological and molecular features of TLSs in autoimmune disease.

Disease	Histological structure	molecules	Cell types	Rate	Function	Refs
RA	Lymphoid type, myeloid type, and fibroid type. TLSs only exist lymphoid type.	IL-6, IL-22, IL-17, CXC-L13, CXCL-12, VCAM-1, BAFF, IL-27	FLS, NK, Th17, FDCs, B cell, NIK+ ECs, Tfh, Tfr	About 40%	Tissue des-truction and fibrosis	(56, 61–73)
**SS**	Autoimmune epithelialitis, lymphocyte accumulation around the glandular ducts, and the appearance of TLSs.	IL-13, TNF-α, LTα1β2, VCAM-1, ICAM-1, IL-22, CXCL-13, CCL19, CCL21, IL-27	Immunofibl-asts, ILCs, T cells, B cells, FDCs, Stro-mal cells, Ductal ECs	30%-40% (FS>1)	Tissue des-truction, fib-rosis, and related to lymphoma	(3, 74–79)
**LN**	Immune complex dep-osition in mesangial and endothelial cells. Chronic inflammatory cell infiltration and formation of TLSs in the interstitium.	BAFF, IFN, IL-23, CXCL12, CCL20, LTα1β2	Ductal ECs, T cells, B cells, FDCs, Tfh,	About 6%	Tissue des-truction and fibrosis	(80–91)
**IgG4-SD**	Cell and extracellular matrix proliferative disease, lymphocyte accumulation, and TLSs formation in inter-stitium.	IL-21, IL-4, IFN-γ, LTβ, LTβR, IL-7, IL-7R, HLA-DRA	Ductal ECs, Th2, Fibro-blast, B cells,	About 60%	Mild tissue destruction and severe fibrosis	(92–96)
**IIM**	A heterogeneous dis-ease, muscle inflame-matory cell infiltration, and muscle fiber necrosis. TLSs preferentially perimysial Localization.	PNAd, CCL21, TNFSF11,	FDCs, B cells, T cells et al.	Low, 20% in JDM	Not clear, tissue injury?	(97–102)
**T1D**	Immune-mediated des-truction of pancreatic beta cells, TLSs formation in the islets.	CXCL13, LTα1β2, CCL19, CXCR5	B cells, T cells, FRCs, FDCs	About 50%	Tissue des-truction, and TLSs gradually disappeared	(103–108)
**MS**	Inflammatory demyel-inating lesions in white matter and TLSs in meninges.	IL-17, IL-21, IL-23, LTβ, IFN-γ	FDCs, Th17, Tfh, Stromal cell network	About40% in SPMS	Tissue des-truction	(109–119)

RA, rheumatoid arthritis; SS, Sjögren’s Syndrome; LN, Lupus nephritis; IgG4-SD, IgG4‐related sialadenitis; IIM, idiopathic inflammatory myopathy; T1D, Type 1 Diabetes; MS, Multiple sclerosis; TLSs, Tertiary lymphoid structures; CXCL13, C-X-C motif chemokine 13; VCAM-1, Vascular Cell Adhesion Molecule 1; BAFF, B-cell Activating factor of the TNF family; FLS, Fibroblast-like synoviocytes; NK, natural killer; FDCs, Follicular dendritic cells; ECs, Epithelial cells; Tfh, Follicular helper T cells; Tfr, Follicular helper regulatory cells; ILCs, Innate lymphoid cells; IFN, Interferon; LT, lymphotoxin; TNFSF11, Tumor necrosis factor ligand superfamily member 11; FS, Focus score; JDM, juvenile dermatomyositis; FRCs, fibroblastic reticular cell; CCL19,   C-C Motif Chemokine Ligand 9; CXCR5, C-X-C chemokine receptor type 5; SPMS, secondary progressive multiple sclerosis.

### 5.1 Rheumatoid arthritis, TLSs in synovial tissue

Rheumatoid arthritis (RA) is the most common autoimmune disease, affecting approximately 1% of the global population (120, 121). In the clinic, patients who suffer persistent disease activity lead to inflammation of multiple joints, progressing to cartilage destruction and bone erosion (122). The pathological nature of RA is synovitis, which is characterized by the infiltration of immune cells (including innate immune cells and lymphocytes), the proliferation of FLS (synovial fibroblasts), and the growth of new blood vessels (61). According to the pattern of synovial biopsies, RA can be divided into three groups, lympho-myeloid (B cell aggregates present), diffuse-myeloid (sublining macrophage infiltration), or pauci-immune fibroid (lack of or low inflammatory cell infiltrate) (62, 63). TLSs can be found in about 40% of untreated RA patients, and they can only be developed in the lympho-myeloid group (64). Besides, stromal cells are equally important in the production and development of TLSs (65). Two synovial cells (fibroblasts-like synoviocytes and macrophages-like synoviocytes) exist in the joint (66). Fibroblast-like synoviocytes (FLS) are mesenchymal origin with various immunomodulatory functions. FLS can secrete IL-6, IL-7, VCAM-1, CCL21, CXCL13, CXCL12, and BAFF, as well as APRIL (66, 67). These regulatory molecules maintain the survival and function of immune cells and make them to be organized more orderly. In addition, FLS also expresses the receptor of IL-22 (68). NK cells and infiltrating immune cells such as Th17 can secrete IL-22 and promote the proliferation of FLS (69). Therefore, under the influence of genetic and environmental factors, synovial local immune tolerance is broken. The interaction between infiltrating immune cells and stromal cells, especially FLS, promotes the formation of local TLSs and affinity maturation and class switching of local autoreactive B cells (70). This evidence suggests that therapies targeting TLSs may be beneficial in part of patients with RA. Animal experiments have demonstrated that IL-27 can negatively regulate the formation of TLSs, as well as increase the proportion of follicular regulatory T cells (Tfr) in ectopic germinal centers, which can inhibit germinal center response (57, 71, 72). However, the results of clinical trials are disappointing. Inhibition of LT-β, IL-17, or IL-12/23 did not show significant efficacy (73, 123). However, RA is a strong heterogeneity of disease, and TLSs only exist in part of patients. Stratification based on synovial pathology is important for optimizing treatment regimens. Future may need to design more rigorous clinical trials for accurate assessment, and more basic research is needed to clarify the critical factors of TLSs in RA.

### 5.2 Sjögren’s syndrome, TLSs in salivary gland

Sjögren’s syndrome (SS) is an autoimmune disease that mainly affects the exocrine glands (salivary and lacrimal glands) (124). Apart from xerostomia and keratoconjunctivitis sicca, SS has multiple systemic manifestations including arthralgia, fatigue, autonomic dysfunction, vasculitis, hyperglobulinemia, hypocomplementemia, as well as renal, skin, nervous system, and lung involvement (125). Among them, approximately 5% can progress to lymphoma (74, 125). Lymphocyte infiltration around the gland ducts is one of the pathological features of SS. TLSs, which are closely related to the activation of immunofibroblasts, have been reported to be found in approximately 30–40% of SS patients, when considering patients who present with a focus score >1 in labial salivary gland biopsy samples (3, 75). IL-13, which is released from resident ILCs, stromal cells, fibroblasts, and epithelial cells, is proven to be crucial for the function of immunofibroblast (76). IL-13 can up-regulate the adhesion molecule VCAM-1 by binding to IL-4R and have a synergistic effect with TNF-α and LTα1β2 to increase the expression of ICAM-1 and podoplanin, which further interacts with infiltrating immune cells (76). At the same time, lymphocytes release IL-22 to promote the proliferation and maturation of immunofibroblasts, which upregulate and release chemokines CXCL13, CCL19, CCL21, etc (54). Furthermore, infiltrating immune cells, stroma-derived FDC, and ductal epithelial cells were also shown to express CXCL13 (3, 50, 76, 77). Under the influence of chemokines, more lymphocytes are recruited and gradually develop into TLSs. It is worth emphasizing that TLSs are associated with higher levels of antibodies and more systemic symptoms (75), and are also associated with a 16-fold increased risk of lymphoma in SS patients (75, 78). Given the importance of TLSs in SS and the efficacy of LTβR blockade in animal models, corresponding clinical trials have been carried out (79, 126). Although not proven effective, probably because the blockade of LT-βR did not completely inhibit lymphocyte recruitment (126). However, we must note the redundant role of cytokines. Only inhibiting one of them makes it difficult to block the progress of the disease. In addition, the enrolled patients were not screened for pathology. Therefore, there is still reason to believe that targeted TLSs are effective, but it is necessary to find out who needs to target TLSs therapy and who will benefit more.

### 5.3 Lupus nephritis, TLSs in kidney

Lupus nephritis (LN) is a common and serious complication of systemic lupus erythematosus (SLE) (127). The most common clinical manifestations were proteinuria and hematuria (80). It has been reported that the aggregation of T and B cells together with the formation of the FDC network occurs in 6% of patients (81). The occurrence of TLS in LN is infrequent, possibly influenced by disease progression, treatment process, biopsy location, and other factors. In mice models of LN, such as NZB/W mice, although the incidence of TLSs is not specified, all mice in the experimental group exhibit TLSs (82). Before the onset of LN, the immune tolerance has been broken, and autoantibodies appear (such as dsDNA). Immune complexes (IC) can be deposited in the kidney to destroy the filtration membrane and activate complement, promoting the development of tissue damage and inflammation (83, 84). Chronic inflammation leads to changes in the function of renal resident cells, and the changes have been reviewed (85). Notably, renal ductal epithelial cells secrete BAFF and type 1 IFN and express the costimulatory molecule B7-H4, which can activate T cells (86–88). In addition, they also produce CXCL12 and CCL20 in response to IL-23 to recruit lymphocytes to the renal mesenchyme (89). It has been shown that the TLSs gene profile in the kidney of lupus-susceptible mice is similar to that in lymph nodes during active LN (90). Moreover, FDC-like structures and PNAd+ HEVs were found in the kidneys of LN patients, and the development of HEVs was closely related to LTβR signaling (81, 91). BAFF is also involved in TLSs in LN, not only by enhancing B-cell chemokine signaling but also by promoting follicular helper T cell (Tfh) activity and prolonging the in-site response in TLSs (128). The present studies support that TLSs can be formed in the mesenchyme of LN and are functional. Although the exact details of TLSs formation and its contribution to disease are not clear, with the continuous improvement and deepening of multi-omics methods, more targets for TLSs can be provided in the future.

### 5.4 IgG4-related dacryoadenitis and sialadenitis, TLSs in salivary gland

Immunoglobulin G4-related disease (IgG4‐RD) is an inflammatory disease that is characterized by elevated serum IgG4, organ enlargement, and tissue fibrosis, which can affect multiple organs, including the meninges, eyes and tear ducts, salivary gland, thyroid gland, the pancreas, and biliary tract, lungs, kidneys and retroperitoneal, etc (129). Of which salivary gland involvement, was previously known as Mikulicz disease, namely IgG4-related dacryoadenitis and sialadenitis (IgG4-DS). IgG4-DS and SS both involve the salivary glands, but there are many differences (130). SS, known as autoimmune epithelitis, is characterized by severe acinar atrophy, whereas IgG4-DS is considered to be a disorder of cellular and extracellular matrix proliferation, characterized by hyperplastic germinal centers and mild acinar atrophy (92, 131). Many immune cells are involved in the formation of TLSs in IgG-DS patients. Among them, the excessive production of IL-21 by Th2 cells is thought to be involved in Bcl6+ B cells in IgG4-DS patients, leading to the massive formation of germinal centers (about 60%) (93, 94). IFN-γ and IL-4 can actively participate in the formation of TLSs in IgG4-DS (95). IFN-γ significantly increased B cell lymphoma 6 (Bcl6), PR domain containing protein (PRDM1), and paired box homeotic gene 5 (PAX5) levels in B cells from IgG-4-DS patients and immortalized B cell lines, while IL-4 could increase the expression of X-Box Binding Protein 1 (XBP1) (95). These suggest that IFN-γ and IL-4 both can influence the capacity of B cell germinal center responses, and promote proinflammatory phenotype and differentiation into plasma cells. Moreover, IFN-γ can significantly increase the levels of human leukocyte antigen DRA (HLA-DRA) in fibroblasts and salivary gland epithelial cells. And interestingly, IFN-γ and IL-4 both can upregulate genes involved in TLSs formation, including LT-β, IL-7R, and LTβR (95). Somatic hypermutation (SHM) and class switch recombination (CSR) occur continuously in the TLSs of IgG4-DS. IgM+ B cells switch isotype to IgG4+ B cells. IgG4+ B cells with mature affinity can recognize autoantigens more effectively, which are thought to be able to act as antigen-presenting cells (APCs) to activate CD4+ cytotoxic T lymphocytes (CTLs) to aggravate tissue damage (95, 96, 132). Unfortunately, IgG4 is absent in mice, resulting in a lack of effective animal models. Although more studies are needed for further support, targeting TLSs may contribute to improving the initiation and progression of IgG-DS.

### 5.5 Idiopathic inflammatory myositis, TLSs in muscle

Idiopathic inflammatory myositis (IIM) is a heterogeneous group of autoimmune myopathies, with an estimated incidence of 0.1-1.0 per 100,000 persons per year (97). Lymphocytic infiltration and TLSs may also be found in IIM (98, 99). However, in patients with polymyositis, typical TLSs in the affected muscle appeared to be uncommon, and the expression of PNAd and CCL21 in the blood vessels was weak (100). Similarly, in idiopathic dermatomyositis (DM), TLSs are also rare (101). DM with TLSs may be a rare variant. Even if the incidence is low, TLSs are still functional, and lymphocytes in muscle and the lymph nodes can recycle, indicating that the former may also produce antibodies (101, 102). Tumor necrosis factor superfamily 11 (TNFSF 11) has also been implicated in the formation of TLSs, and TLSs are associated with patients who are more severe and difficult to treat (4, 101). Sporadic inclusion body myositis (sIBM) also found TLSs, but the specific meaning is unclear (133). In juvenile dermatomyositis, TLSs can be found in approximately 20% of patients and are preferentially localized to the perimysium. B cells in TLSs can undergo clonal diversification and differentiate into plasma cells (98). In addition, immune checkpoint inhibitors (such as PD-1 inhibitors) may also lead to muscle damage and form TLSs, in which CD21+ FDCs, and PNAd+/CCL21+ HEV both exist (100). Overall, although the rate of TLSs in autoimmune myositis is not high, antigen-driven humoral response can still be observed. Future studies are needed to clarify whether targeting TLSs has advantages in the treatment of refractory myositis.

### 5.6 Type 1 diabetes, TLSs in the pancreas

Type 1 diabetes (T1D) is a common, organ-specific autoimmune disease that is mediated by T cells responding to self-antigens in the pancreatic β-cell (134). Patients with T1D commonly present with symptoms of polyuria, polydipsia, and weight loss, and approximately a third present with diabetic ketoacidosis (103). In mouse models of type 1 diabetes (NOD), TLSs can be found in islets, and antigen-driven SHM has also been proven to occur in them (104). Moreover, the repertoire of Ig VL chain genes in infiltrating B cells is highly diverse and differs from that observed in regional lymph nodes (104). It has been reported that blocking lymphotoxin can prevent the development of diabetes in NOD mice (105, 106). However, only blocking CXCL13 disrupted B cell recruitment, and the diabetes still progressed normally (107). It is also interesting to note that TLSs exist early during the disease and can promote disease progression by inducing adenosine deaminase (AID), producing autoreactive CD138+ plasma cells, but they are significantly reduced in aged NOD mice, supporting evidence that TLSs in T1D are associated with a more aggressive phenotype and higher pathogenicity (108). Similarly, TLSs are present in 50% of human type 1 diabetes in preclinical and clinical settings, in which HEV, reticular cells, and abundant immune cell subtypes can be detected (135). Overall, patients of TID with TLSs are younger compared to those lacking TLSs, and with the aggravation of β cells, the density of TLSs gradually decreases, which is similar to the results in NOD mice (135). These results suggest that TLSs exist in the early stage of the disease and generate autoreactive T and B cells to destroy β cells. When the target antigen is exhausted, TLSs disappear. These results further support the TLSs on the progress of TID, early targeted therapy will have a positive significance.

### 5.7 Multiple sclerosis, TLSs in the meninges

Multiple sclerosis (MS) is an autoimmune disease characterized by white matter demyelination in the central nervous system (CNS), which mainly involves periventricular white matter, optic nerve, spinal cord, brain stem, and cerebellum (109). Ectopic lymphoid follicles have been reported to be found in the meninges of approximately 40% of the post-mortem brain tissue in progressive multiple sclerosis (MS) and are associated with the severity of cortical degeneration and clinical disease progression (110). However, there are few studies about TLSs in MS patients, which may depend on the difficulty of obtaining meninges and the complexity of treatment (111). Gardner et al. found elevated levels of the proinflammatory cytokines including IFN-γ and TNF-a in the meninges of secondary progressive multiple sclerosis (SPMS) cases with TLSs (112). Serafini et al. found in the SPMS patients, there exists interleukin-17 (IL-17) secreting cells and RORγ+ cells in TLSs (113). These cells may contribute to the interaction between B cells and Tfh, which is helpful to meningeal lymphogenesis. The experimental autoimmune encephalomyelitis (EAE) model of mice can mimic MS. In the EAE model induced by PLP139-151, treatment with lymphotoxin β receptor-Ig fusion protein not only inhibited TLSs formation but also improved the symptoms of EAE (114). In the model of adoptive transfer Th cells, only Th17 cells were able to induce TLSs formation in recipient mice (115). In another study, the depletion of IL-21R and IL-23R reduced the severity of EAE and the number of TLSs in mice (116). Tfh is also involved in the TLSs reaction and has been suggested to contribute to the maintenance of TLSs (117, 118). However, the specific role of Tfh in MS is still unknown, and there are some contradictions (119, 136). These results demonstrate the pathogenicity of TLSs, but further exploration is needed to explore the effectiveness of TLSs-targeted therapy and the details of TLSs formation.

## 6 Discussion

TLSs can be found in autoimmune diseases, chronic inflammation, organ transplantation, and tumors (4, 6), of which inflammation is a common feature in the pre-symptomatic stages of these diseases. Inflammation means that tissue local postcapillary venules upregulate inflammation-related adhesion alterations and thus recruit the initial leukocytes. Under normal circumstances, pro-inflammatory cells gradually subside with the removal of the pathogens and local activation of lymphocytes choose apoptosis via AICD. However, when the pathogen cannot be completely removed, or autoantigens are continuously exposed to activated lymphocytes, these cells have the opportunity to survive for a long time, which can act as LTi by secreting lymphotoxin. After long-term interaction with stromal cells or endothelial cells, stromal cells such as fibroblasts can secrete CXCL13, CCL19, CCL21, etc. to recruit and compartment local T and B cells, while endothelial cells can further express HEV-related properties, such as up-regulation of the chemokine CCL21, recruitment of naive and memory lymphocytes to local tissues. TLSs have also been proven to be functional, which can express AID, inducing SHM and CSR (7). Therefore, TLSs are associated with an aggressive phenotype in autoimmune disease. There are several possible outcomes for TLSs. Firstly, TLSs decrease and vanish gradually with the gradual depletion of target antigens. Secondly, TLSs may participate in the fibrosis of local tissues. Thirdly, TLSs may be related to the local occurrence of mucosa-associated lymphoma.

However, the exact process of TLSs initiation and formation is still not fully understood. What is the relationship between TLSs and the draining lymph nodes? How do the lymphocytes of TLSs circulate? Can memory lymphocytes in TLSs re-enter the lymphatic circulation? Can immune tolerance in TLSs be induced by altering the local cytokine microenvironment? What is the classification and function (support, nutrition, and compartmentalization) of TLSs-related stromal cells? In addition, it is also worth noting that autoimmune disease-related TLSs have not been stratified based on pathology. Further study will be required to better understand the risk stratification of TLSs based on pathology. To investigate the association of functional TLSs or secondary follicle-like TLSs with the severity or antibody titer of these diseases and the response to treatment.

## 7 Conclusions

In general, TLSs are an adaptive defense against persistent failure to relieve inflammation. These are thought to be associated with a better prognosis in tumors and increase the therapeutic effects of immune checkpoint inhibitors (ICIs) (5). For autoimmune diseases, these are often related to tissue damage and fibrosis (3). Therefore, targeted TLSs are thought to improve the prognosis of autoimmune diseases, and according to the pathology choosing the appropriate cytokines to inhibit the formation of TLSs may be more effective. We believe that over the next few years, there will be more exciting discoveries, which can provide more details and options for the targeted TLSs treatment.

## Author contributions

YD: Data curation, Investigation, Writing – original draft, Writing – review & editing. TW: Data curation, Writing – original draft, Writing – review & editing. HW: Methodology, Supervision, Validation, Writing – review & editing.
